# Y-Box Binding Proteins in mRNP Assembly, Translation, and Stability Control

**DOI:** 10.3390/biom10040591

**Published:** 2020-04-11

**Authors:** Daria Mordovkina, Dmitry N. Lyabin, Egor A. Smolin, Ekaterina M. Sogorina, Lev P. Ovchinnikov, Irina Eliseeva

**Affiliations:** Institute of Protein Research, Russian Academy of Sciences, Pushchino 142290, Russia

**Keywords:** YB-1, YBX1, YB-2, YB-3, Y-box-binding proteins, mRNP, RNA, translation regulation, stability regulation

## Abstract

Y-box binding proteins (YB proteins) are DNA/RNA-binding proteins belonging to a large family of proteins with the cold shock domain. Functionally, these proteins are known to be the most diverse, although the literature hardly offers any molecular mechanisms governing their activities in the cell, tissue, or the whole organism. This review describes the involvement of YB proteins in RNA-dependent processes, such as mRNA packaging into mRNPs, mRNA translation, and mRNA stabilization. In addition, recent data on the structural peculiarities of YB proteins underlying their interactions with nucleic acids are discussed.

## 1. Introduction

Y-box binding proteins (YB proteins) were first identified as DNA-binding proteins involved in the regulation of transcription in various organisms [1]; their major property was thought to be the ability to bind to double-stranded DNAs containing the so-called Y-box motif. These proteins are known to be studied previously as major universal components of messenger ribonucleoprotein particles (mRNPs) in various organisms and cells. The sequencing of *Xenopus laevis*, murine, and rabbit RNA-binding proteins evidenced their identity [1]. Later, not only mammalian YB proteins but also those from fish [2,3], mollusks [4], and insects [5] were sequenced and cloned. 

An analysis of amino acid sequences of YB proteins revealed their membership of a broader family of proteins containing a cold shock domain, which is structurally and functionally close to prokaryotic cold shock proteins. To date, the best-studied YB protein is YB-1. The studies of this protein have largely contributed to the recognition of the wide functional abilities of YB proteins. YB-1 is involved in cell differentiation, embryonal development, and stress response. Its role in malignant cell transformation and inflammation is also under intensive study. The putative mechanisms of YB-1 impact on cellular events are numerous; they imply YB-1 involvement in virtually all DNA- and RNA-associated processes and depend on its extracellular or intracellular localization and modification. 

Since one review can hardly cover all the proven and alleged roles of YB proteins in a cell or the whole organism, here we focus on peculiar interactions of these proteins with RNAs and their involvement in RNA-dependent processes, such as mRNA packaging into mRNPs, mRNA translation, and mRNA stabilization. Other no less interesting activities of YB proteins are described in several comprehensive reviews [1,6,7,8,9,10,11].

## 2. The Structure of YB Proteins

**Figure 1:**
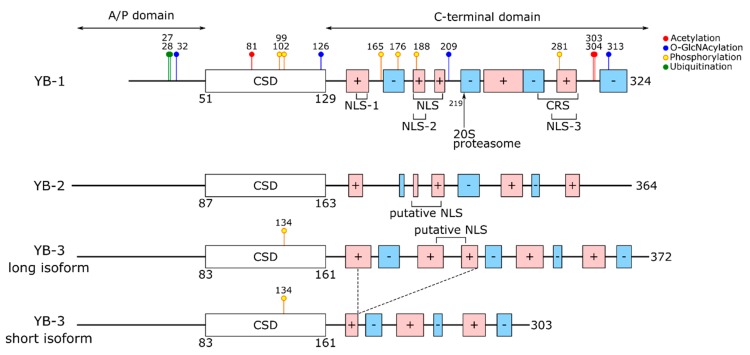
Domain organization of Y-box binding proteins (YB proteins). A/P domain, alanine/proline-rich domain; CSD, cold-shock domain; NLS, nuclear localization signal; CRS, cytoplasmic retention signal. Clusters of positively and negatively charged amino acid residues are shown in red and blue, respectively. Modifications of YB proteins are indicated here and presented in Table 1 (see below).

In YB proteins, the cold shock domain (CSD) is flanked by the N-terminal alanine/proline-rich (A/P) domain and the extended C-terminal domain (CTD) containing positively and negatively charged clusters of amino acids (Figure 1) [1]. In all three members of the family, cold shock domains show more than 90% identity, while C-terminal domains are close in amino acid composition (Arg, Pro, Glu, Gln, Gly amount to about 60% of all CTD residues) and distribution of charged clusters. The cold shock domains of YB proteins from different organisms are also highly homologous (vertebrate CSDs are approximately 80% identical to those of insects (Yps, *Drosophila melanogaster*); the identity of CSDs from different classes of the vertebrates exceeds 90%). C-terminal domains of YB protein family members from different vertebrates show high homology (approximately 60% identity), while virtually no homology was observed between vertebrate and invertebrate CTDs. Nonetheless, alternation of the clusters of positively and negatively charged amino acid residues is preserved. In contrast, the N-terminal domains of YB-1, YB-2, and YB-3 are the least homologous, although all of them are rich in proline and especially alanine.

According to [8], there are short spacers (called NC9 and CC13) between the domains, although NC9 can be regarded as a part of CSD because it belongs to the β1-strand [12,13].

The YB-1 cold shock domain is currently the only domain of YB proteins with a known spatial structure, which was first determined by NMR [12]. Recently, Yang and colleagues [13] managed to perform an X-ray structural analysis of the complex between the cold shock domain and an RNA fragment. As was expected due to the high identity (approximately 42%) of the YB-1 CSD to the *Escherichia coli* CspA protein, the spatial structure of the mammalian YB-1 cold shock domain is very close to that of major bacterial cold shock proteins. Since the amino acid sequence of YB-1 CSD is almost fully identical to that of YB-2 and YB-3 CSDs, the identity of their spatial structures can also be expected. 

The YB-1 cold shock domain consists of five anti-parallel β-strands forming a β-barrel. The NMR analysis showed low stability of CSD that remains only 70% native at 25 °C [14]. This fact is also supported by microcalorimetry data. As reported by Guryanov and colleagues [15], the mid-point temperature of the YB-1 CSD transition from the native to a denatured state is 35 °C, whereas that of *E. coli* CspA is much higher (56 °C) [16]. This difference is thought to be due to a long mobile loop in the YB-1 cold shock domain and a short one in prokaryotic proteins. Importantly, the aromatic amino acid residues of β1–β3 strands are located on the surface of CSD proteins, thereby presumably stabilizing their structure (in contrast to the usual destabilizing effect of hydrophobic residues), because their replacement by other amino acids, both polar and non-polar, entails lower total stability of CSD proteins [17,18,19]. The exact cause of such an effect is unknown, but a key role is believed to be played by hydrophobic interactions of the aromatic residues, which are disturbed by the introduction of other residues.

The presence of the cooperative tertiary structure is confirmed by differential scanning microcalorimetry for the cold shock domain only, while bioinformatics analysis and circular dichroism (CD) spectroscopy revealed no regular secondary structure in the other two domains. In addition, CD spectroscopy showed the presence of a considerable portion of the polyproline helix type II [15] that is typical of intrinsically disordered proteins and participates in the intermolecular recognition [20].

Of note, many RNA-binding proteins, when unbound, are mostly disordered [21,22,23]. For example, ribosomal proteins and proteins of RNA-containing viruses [24,25] acquire their structure upon RNA binding. Similarly, YB proteins might become structurally arranged when binding to RNA or protein partners; yet so far, the literature offers no such information. 

**Figure 2:**
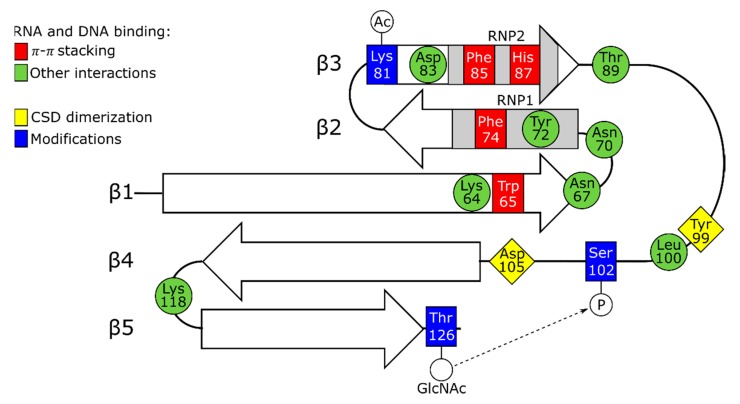
The YB-1 cold shock domain (51–129). Amino acid residues involved in CSD dimerization or nucleic acids binding (π–π stacking and other interactions) are shown in yellow, red, and green, respectively. The RNP1 and 2 consensuses are gray. Modified amino acid residues are blue. Ac, acetylation; GlNAc, O-GlcNAcylation; P, phosphorylation. The effect of GlNAc-Thr126 on Ser102 phosphorylation is represented.

Nonetheless, in solution, molecules of full-length YB-1 are compact and demonstrate the sedimentation coefficient typical of globular proteins [15]. Depending on conditions (primarily, ionic strength) and concentration, YB-1 can form various oligomers (from 3 to 20 molecules) [15]. Importantly, the CSD and A/P-CSD fragments of YB-1 show no such compactness and oligomerization ability, and the last half of the C-terminal domain is prone to aggregation. Hence, in YB-1 oligomerization, the crucial role is played by the C-terminal domain. This domain was also shown to be responsible for the multimerization of FRGY1 and 2 [26]. A recent study [13] demonstrates that cold shock domains can form dimers using Tyr99 and Asp105 of the long loop between the β3- and β4-strand (Figure 2). Therefore, CSD assistance in the oligomerization of YB proteins cannot be ruled out. 

## 3. Interaction of YB Proteins with Nucleic Acids

### 3.1. The Role of Cold Shock Domain

Similar to prokaryotic proteins, the YB-1 CSD comprises two consensus motifs RNP1 and RNP2 localized to the β2-strand and β3-strand, respectively. Likewise to bacterial proteins with the cold shock domain (Csp), the aromatic amino acid residues of these motifs Phe74, Phe85, and His87 are located on the protein surface, thereby forming a hydrophobic cluster, and they participate in nucleic acid binding [12,13]. These residues are functionally close to Trp65, although the latter resides in the β1-strand and belongs to no motif. Interestingly, the CSD residue Tyr72, corresponding to CspA Phe18, is uninvolved in the π–π interaction with nucleic acids (X-ray data) and remains unobservable for NMR. When interacting with RNA oligos, the hydrophobic residues His87, Phe85, Phe74, and Trp65 form π–π-stacking pairs with four RNA bases (Figure 2). These residues are most conserved and have been detected in both prokaryotic and eukaryotic cold shock domains [8]. The replacement of any of them with Ala leads to the disruption of interaction with the RNA oligos [13]. The integrity of the CSD structure should be strictly controlled because the Phe74Leu substitution was shown to disrupt the stacking of β-strands [27], and in prokaryotic Csp, such substitutions entail a crucial effect on protein stability [18,19].

Apart from the above hydrophobic amino acids, the CSD-to-nucleic acid binding is mediated by Arg69 and Lys118 (NMR data) located between the β1–β2 and β4–β5 strands, respectively [12]. The crystallographic analysis [13] of the CSD complex with a CAUC-containing RNA oligo revealed a net of intermolecular hydrogen bonds (H-bonds) between C_1_ and the residues Thr89 and His87, as well as between the C_1_ ribose 2’ hydroxyl group and Lys118. The latter not only forms a salt bridge and is H-bonded to the A_2_ phosphate group, but it stacks over A_2_ to buttress the cation–π–π sandwich structure, and it also mediates the H-bonding of N^6^ in A_2_. It is not surprising that the replacement of Lys118 with Ala inhibits the RNA binding completely. Lys64 is another important residue mediating RNA interactions whose replacement entails a complete blockage of the RNA binding. Through H-bonding, this residue interacts with O^2^ of the third base (U_3_), and forming a salt bridge to Asp83, it binds to N^3^ of U_3._ In the interaction with C_4_, an important role is played by Trp65 stacking, as well as H-bonds occurring between Trp65, Asn67, Asp105, Asn70, and Tyr72 (Figure 2). Together, the crystallography data show that additional H-bonding and extra salt bridges can determine the specificity of the CSD interaction with RNA. As reported by Yang and colleagues, the CAUC motif shows a higher affinity for the YB-1 CSD because it provides numerous additional bonds [13].

As mentioned above, eukaryotic and prokaryotic cold shock domains are structurally very close, except for a longer loop between the YB-1 CSD β3 and β4 strands. According to NMR data, Leu100 residing in this loop and surrounded by three positively charged residues and one aromatic residue showed a reliable change in the chemical shift after YB-1 CSD binding to a DNA oligo, which indicates the involvement of this loop in the interaction with nucleic acids [12]. Furthermore, the replacement of the short loop of prokaryotic CspA CSD by the long loop of YB-1 CSD enabled CspA to bind double-stranded DNA (dsDNA) in addition to single-stranded DNA (ssDNA) [27]. Interestingly, in CspA, Phe20 substitution by Leu (Phe20 corresponds to Phe74 in YB-1 CSD) decreases its affinity for ssDNA, but with the short loop replaced by the long loop from YB-1, the modification Phe20Leu produces only a slight effect on the CspA interaction with ssDNA [27]. This also indicates that DNA binding and probably RNA binding is mediated not solely by the hydrophobic surface formed by the β1–β3 strands but by other residues beyond it.

### 3.2. The Role of the C-Terminal Domain

It is hardly possible to describe the interaction of the C-terminal domain with nucleic acids because the spatial structures of the full-length YB-1 and YB-1 CTD in complex with nucleic acids are unknown. However, the presence of the C-terminal domain increases CSD affinity for both RNA and DNA [28,29]. It was suggested that the interaction between CTD and nucleic acids is underlain by electrostatic interactions of positively charged amino acid residues abundantly present in CTD with the sugar–phosphate backbone of RNA or DNA [30]. It cannot be ruled out that the affinity for nucleic acids grows due to the CTD ability to promote protein multimerization [26,31] that brings several nucleic acid binding sites closer to one another. Currently, the literature offers only one study of the spatial structure of the complex between 30-nt RNA/ssDNA and the YB-1 fragment (1–180) containing CTD residues [32]. The authors report that YB-1 (1–180) in complex with RNA/ssDNA forms extensive filaments, but when in complex with 30-nt RNA/ssDNA, some of the amino acid residues of the range 130–156 (the first positively charged cluster), but not 166–180, demonstrate a larger chemical shift (NMR data). In addition, in this range, a molecular modeling analysis revealed an interaction between the arginines of one molecule and RNA/ssDNA phosphates at the surface of a nearby cold shock domain. This suggests that during RNA interaction with YB-1 (1–180), the C-terminal domain neutralizes phosphates of the DNA/RNA sequence closest to the cold shock domain of the adjacent 1–180 molecule, which disrupts folded nucleic acid secondary structures to allow the formation of the linear filament. Full-length YB-1 in complex with RNA is incapable of forming such filaments presumably because the tail portion of CTD neutralizes phosphates of more numerous nucleic acids, thereby preventing the interaction between the proximal CTD sequence and the cold shock domain of the adjacent 1–180 molecule [32].

### 3.3. In Vitro Interactions of YB Proteins with Nucleic Acids

Initially, the preferential binding partner of YB proteins was believed to be dsDNA containing the Y-box motif [26,33,34,35,36,37,38]. Later studies reported that YB proteins favor pyrimidine-rich fragments of dsDNA [39,40,41,42], DNA containing apurinic sites [43,44], and ssDNA [39,41]. Finally, after YB proteins have been identified as the major component of mRNPs, their RNA binding ability became evident [38,45,46,47]. This could not but raise the following questions:

Do YB proteins have a higher affinity for DNA or RNA? 

Do YB proteins bind specific nucleic acid sequences or motifs? 

What regions or domains of YB proteins contribute to binding specificity?

Many researchers report that the affinity of these proteins for RNA is higher than for ssDNA (Kd approximately 10^−8^ for RNA [48,49,50] and 10^−6^ for DNA [51]). Some YB proteins were shown to display a notably lower affinity for dsRNA or none at all [13,52]. Lastly, there is decisive evidence for the much higher affinity of YB proteins for ssDNA as compared to dsDNA [12,29,40,43,49,53]. In YB proteins, CSD and CTD are responsible for DNA and RNA binding. Specifically, the isolated C-terminal domain or its fragments are incapable of DNA binding but quite efficient in RNA binding [26,29,49]. CSD is required for both RNA and DNA binding. Interestingly, CTD (and even solely its first half) promotes interaction between CSD and RNA [29]. These findings are partially supported by ITC (isothermal titration calorimetry) data [51]. Moreover, molecular dynamics has demonstrated a higher affinity of the cold shock domain for RNA than DNA [54].

The specificity of YB proteins is a challenge. Initially, these proteins were thought to specifically bind Y-box-containing DNA, as was confirmed by many experiments on Y-box-containing DNA oligos (see, e.g., [55,56]). However, almost concurrent studies demonstrated the ability of YB proteins to interact with DNA fragments free of the Y-box motif [40,42,43,57] but containing pyrimidine-rich sequences. An analysis of the specificity of rabbit YB-1 binding to ssDNA and dsDNA fragments revealed a wide variety of bound sequences in the following order of preference: the single-stranded GGGG motif > the single-stranded and double-stranded CACC and CATC motifs > the Y-box motif (moderate affinity) [58].

Interestingly, the interaction between YB proteins and DNA strongly depends on the integrity of the DNA secondary structure [29,43,44,59,60]. In addition, as shown recently by atomic force microscopy (AFM), YB-1 binds preferentially to supercoiled DNA at DNA crosses [61].

YB-1 and its closest homologs can interact with any RNA sequence, although A- and C-rich sites and the CANC motif are somewhat more preferable [1,28,62,63,64,65,66]. Therefore, Yang and colleagues [13] performed the crystallization of the YB-1 cold shock domain in complex with a specific CAUC-containing sequence. Their study shows that RNA fragments with this motif display 2 to 3 times higher affinity for YB-1 than non-specific RNA fragments, which is consistent with the literature data [67]. The ability of YB-1 to specifically interact with the 5′-tiRNA^Ala^ (probably with oligoguanine motifs at their 5′-ends) [68] is somewhat out of the general specificity typical of YB proteins but coincides with both the results of studies using DNA-carrying microchips and experiments on RNA homopolymers [48,58].

It is not a recent finding that the cold shock domain of YB proteins determines the specificity of their interactions with nucleic acids, while CTD and the A/P domain support this process [26,28]. The C-terminal domain appears to determine the non-specific binding almost exclusively through contacts with the RNA sugar–phosphate backbone [30]. However, as in some other RNA-binding proteins (RBP), in YB proteins, the CTD arginine-rich motifs (ARM) are also able to determine the specificity of nucleic acid binding [8,69]. Importantly, the RNA binding specificity of the two domains can depend on varying outer parameters, such as ionic strength or Mg^2+^ or heparin concentrations [51,69]. An elevated Mg^2+^ concentration suppresses the RNA-to-CSD binding, while an increased heparin concentration down-regulates the C-terminal domain binding. In addition, these parameters affect the protein specificity in RNA binding [69]. An increased ionic strength decreases the constant of RNA binding to the full-length protein but produces no effect on the CSD-to-RNA binding [51]. In total, the interactions of these two domains with RNA are controlled by different forces. Moreover, in studying the binding specificity of YB proteins, due attention should be paid to the RNA length. With a length below 30 nucleotides, the FRGY2 affinity for this RNA notably decreases [28]. It can be speculated that not the entire C-terminal domain is involved in binding approximately 30 nt RNAs because longer RNAs would require additional interactions with CTD.

Lastly, difficulties in the specificity determination of YB proteins can partially depend on their ability to melt the secondary structure of nucleic acids [46,70]. In this respect, they are similar to prokaryotic Csp proteins [71], which indicates the key role of CSD in secondary structure melting. Interestingly, in contrast to RNA helicases that use ATP hydrolysis to unwind RNAs, neither YB-1 nor Csp requires any additional energy source for the melting activity, although the detailed mechanism of this process remains unknown. The microchip-using experiments have shown that rabbit YB-1 is most efficient in the destabilization of double-stranded DNAs containing the GGTG, GATG, and GTGG motifs, while duplexes with GGGGG showed a strong YB-1-induced stabilization [58]. It was also demonstrated that YB-1 can accelerate the renaturation of double-stranded DNAs by many thousand-fold in physiological conditions [70]. When RNA-bound, YB-1 melts the RNA secondary structure, although not completely (for α-globin mRNA, as much as 60% at 20 °C) [46]. Whether YB proteins show their melting or stabilizing activity, presumably strongly, depends on the protein/nucleic acid ratio, as well as on the nucleotide composition and length of the complementary chain [70]. Hence, the binding ability of these proteins can be different for different nucleic acid sequences and produce different effects on their spatial structures. Moreover, it has been shown at least for YB-1 that it associates with RNA helicases, which probably affects both its specificity to mRNAs and the regulation of translation of these mRNAs [72,73,74].

Of note, unlike cold shock domains of YB-1 and YB-3, the YB-2 CSD has Phe 99 instead of Tyr99. The same preference for Phe is demonstrated by the YB-2 C-terminal domain. This may well affect the RNA-binding properties of YB-2 [8].

### 3.4. In Vivo Interactions of YB Proteins with Nucleic Acids

The interaction of YB proteins with cellular mRNAs on the scale of the entire transcriptome has become eligible for discussion only after the development of high-throughput methods. The first such data were on the immunoprecipitation of cell-expressed HA-YB-1, followed by the microchip-based analysis [75]. It was reported that HA-YB-1 binds only 20% of cellular mRNAs. One of the earliest CLIP-seq (crosslinking and immunoprecipitation followed by high-throughput sequencing) experiments on breast cancer MDA-231 cells revealed more than 4000 endogenous transcripts bound by YB-1 [76]. However, recent iCLIP (individual-nucleotide resolution CLIP) experiments on glioblastoma cells have demonstrated the YB-1 binding to about 15,000 RNAs, which is commensurable with a whole transcriptome [77]. For YB-3, eCLIP (enhanced CLIP) showed that it binds a broad set of specific RNAs without a discernible binding motif (8727 binding regions within 4018 RNAs) in HeLa cells [78]. In addition, the RIP-Seq (RNA Immunoprecipitation Sequencing) analysis showed that in HEK293T cells, the mRNA sets bound to YB-1 and YB-3 are almost the same, and they amount to about 80% of the transcriptome [79]. Yet, the specificity of these proteins is somewhat different.

As concerns YB-2, the studies of its RNA targets were restricted to germ cells to which it shows specificity. The literature offers no data on the entire range of YB-2-interacting RNAs, although YB-2 and YB-3 are known to modulate the translation of many mRNAs of the testis, which suggests a wide range of targets [80]. The elevated affinity of YB-2 and YB-3 for mRNAs containing YRS (Y-box protein recognition sequence [28]) is indirectly supported by the fact that the translation inhibition depends on the YRS presence in conditions of a limited YB protein amount [80].

The iCLIP technique allowed identifying the preferential YB-1-to-RNA binding site UC/UAuC (UYAUC) mostly located in coding regions and 3′UTRs [77]. As found, this binding site is present in about 18% of all bound RNAs, which suggests that the rest of the RNAs are bound to YB-1 non-specifically. Furthermore, these iCLIP experiments have demonstrated the ability of YB-1 to bind miRNAs, non-coding RNAs, and pre-mRNAs. Interestingly, PAR-CLIP experiments on several cell lines using the overexpressed Flag-YB-1 [81] revealed the same specific YB-1-binding motif CAUC, although it was necessarily preceded by UCUUU. However, the binding motif analysis reported in this study shows that this site is rather degenerate and should probably contain only a CANc motif. In addition, methodically similar studies by Gopanenko and colleagues [82] revealed for YB-1 a somewhat different major motif, as well as many other motifs, thereby suggesting that for YB-1, there are degenerate consensus sequences that are mostly located in 3′UTRs. A peculiar PAR-CLIP data processing and overexpressed Flag-YB-1 used in this work could affect the results because the protein amount exceeding the normal level entails non-specific RNA binding, thus causing a “smeared” specific motif. Moreover, the use of antibodies specific not to YB proteins themselves but their tags can largely contribute to the erroneous interpretation of experimental results [83].

Importantly, according to [84], YB-1 interacts with ACCAGCCU, CAGUGAGC, and UAAUCCCA, which are potential motifs [85] required for RNA sorting into exosomes. Another protein interacting with one of these motifs is m^5^C-RNA methyltransferase NSUN2, and as shown recently, zebrafish (zYB-1) and *Drosophila* (YPS) YB-1s exhibit elevated affinity for m^5^C-methylated mRNA [86,87]. 

For YB proteins shown to interact with RNA in the cell, photo-cross-linking and high-resolution mass spectrometry analyses revealed only RNA cross-linking with peptides containing RNA consensus I and II from the cold shock domain β-strands 2 and 3 [88]. This could provide evidence that in mRNPs, solely CSD interacts with RNA, while the C-terminal domain is involved in the multimerization of YB proteins or binding other RBPs. It cannot be ignored that mass spectrometry sample preparation implies the usage of trypsin that is specific to Arg and Lys widely available in CTD; this protease produces too short peptides to be identified as fragments of YB proteins. 

The interaction of YB-1 with DNA was studied by ChIP-on-chip (Chromatin immunoprecipitation followed by DNA microarray) and ChIP-Seq (Chromatin immunoprecipitation followed by sequencing) [89,90]. A detailed analysis of these data by Dolfini and Mantovani [91,92] refuted the common conviction of many years that the Y-box motif (CTGATTGGT/CT/C) is the specific binding site of YB proteins. More advanced bioinformatics methods than those used previously [89,90] failed to detect a pronounced YB-1 binding motif. The absence of such a motif is very atypical for the “classical” transcription factor. Furthermore, the YB-1 precipitation with chromatin is probably caused by YB-1 binding to nascent RNAs: ct-YB-1 (*Chironomus tentans* YB-1) lost its association with polytene chromosomes upon RNase treatment [93]. Tying in these data with the stronger RNA binding of YB proteins rather than DNA binding suggests that YB proteins primarily regulate RNA-centric processes.

## 4. Involvement of YB Proteins in the mRNP Formation

As mentioned above, YB proteins were identified as major components of translated and untranslated mRNPs and as the sole proteins of the latter. It was speculated that YB-1 alone is sufficient for the formation of mRNPs with physicochemical properties of native untranslated mRNPs, such as globin mRNA-containing mRNPs from rabbit reticulocytes or mRNPs from *X. laevis* oocytes. Indeed, according to [31], at a high YB-1/RNA ratio, YB-1 molecules interact with mRNA via the cold shock domain, while C-terminal domains are displaced from mRNA and undergo oligomerization, thus forming a large multimeric complex with a sedimentation coefficient and buoyant density identical to those of native mRNPs. Strictly speaking, the reported results do not rule out that not solely CSD but also the adjacent part of CTD (at least up to 198 a.a.) interact with mRNA, forming a complex of about 15–18 protein molecules with a diameter of approximately 20 nm and a height of approximately 7 nm that accommodates at its surface an mRNA sequence of about 600–700 nucleotides [31]. In such a complex, mRNA ends remain inaccessible for translation initiation machinery proteins and exonucleases. At a low YB-1/RNA ratio, YB-1 binds to mRNA as a monomer through the RNA-binding domains CSD and CTD, which results in mRNA unfolding and presumably makes it accessible for the translation machinery. Thus, by mRNA packaging, YB-1 can control the translational status of mRNA and its life span in the cell (see below).

However, the situation when YB proteins are the sole or major proteins of mRNPs is rather rare and occurs only in systems where they are abundant (oocytes, testis, and reticulocytes). The protein composition of mRNPs can also be affected by the isolation procedures. In high salt conditions, many proteins dissociate from mRNAs, whereas YB proteins retain a rather high affinity for mRNAs [94]. Their amount can even increase due to many vacant binding sites on the mRNA. As calculated in [61], the entire amount of YB-1 in the cell would be sufficient to completely package only a small fraction of mRNA molecules. It can be assumed that YB-1 participates in the formation of untranslated mRNPs due to multimerization occurring only on specific mRNAs [61], while on the others, its amount is lower. Alternatively, although present in large amounts [95], YB proteins are far from being the only participants of the packaging of both translated and untranslated mRNPs. As shown, mRNPs can include as many as 860 RNA-binding proteins [96]. Although not all of these are abundant, they still can influence the packaging and the translational ability of mRNPs.

Presumably, YB proteins can be involved not only in the formation of RNPs assembled on mRNAs, but also other RNA-containing particles, such as RNA granules (stress granules, etc.) [97], miRNA-containing complexes for the transport to exosomes [98], and spliceosomes [99].

## 5. YB Proteins in Translational Control

### 5.1. YB Proteins in Translation Inhibition

YB proteins play a dual role in translation, depending on their concentration, certain mRNAs, protein or RNA-partners, and, possibly, modifications. Initially, YB-1 and other YB proteins were identified as major mRNP proteins that packaged and stabilized mRNAs [1,100]. With a high YB-1/RNA ratio, YB-1 inhibits translation by packaging mRNAs into compact untranslated mRNPs where the cap structure and the poly(A)-tail are inaccessible for the poly(A)-binding protein (PABP) and translation factors [100,101]. In these particles, mRNAs are extremely sensitive to endonucleases and tend to acquire surface localization [46], which probably allows regulatory RBPs or regulatory RNAs to recognize mRNAs in untranslated mRNPs and direct them to translation, degradation, or correct location at the proper time or condition. This mechanism of global translational control is extremely important in germ cells and early embryo development [73]. YB proteins are known to be present in abundance in germ cells [73], where they inhibit the translation of dozens of mRNAs [80]. Moreover, translation inhibition, but not mRNA packaging itself, is likely to be most crucial in the maternal-to-zygote transition. As shown, the zYB-1 knockout phenotype can be overcome by overexpression of the 4E-binding protein that inhibits total translation [102]. On the other hand, despite the severe embryonic malformations and fetal death of YB-1 knockout mice, fibroblast cells isolated from YB-1^-/-^ embryos show no significant changes in the global translation [103]. Probably other YB proteins, such as YB-2 and YB-3, or other RBPs functionally compensate for the YB-1 loss. This hypothesis is supported by the observation of even more severe malformations and an earlier death of the YB-1 and MSY4 (mouse YB-3) double knockout embryos [104], as well as by the recent discovery of the functional interchangeability of YB-1 and YB-3 in HEK293T cells [79].

For a long time, doubts were cast upon the role of YB proteins in the global translation inhibition in somatic cells. The recent development of high-throughput techniques allowed demonstrating a decrease in global translation resulting from YB-1 or YB-3 overexpression [79]. Moreover, the magnitude of translation inhibition correlates with the efficiency of YB-1 or YB-3 binding to mRNAs, thus indicating the direct impact of YB proteins [79]. However, the decrease in global translation does not exceed 20%, probably because not all mRNAs are packaged into untranslated mRNPs even with overexpressed YB proteins. Indeed, the estimated YB-1 amount in somatic cells is 5 to 10 molecules per mRNA molecule [105]. Since the medium length of human mRNA is about 3500 nt [106], 75 to 90 YB-1 molecules per mRNA would be required to form an untranslated mRNP. As this amount of YB-1 cannot result even from its overexpression, only a fraction of mRNAs undergo packaging and inhibition by this mechanism in somatic cells. Of note, this mechanism of YB-1-mediated packaging of certain mRNAs into mRNPs has been proposed quite recently [61]. Since YB-1 binds RNA cooperatively, once formed, the mRNP complex remains stable, and free mRNAs do not compete for binding to YB-1 recruited to this complex. In other words, there are YB-1-saturated mRNAs packaged into mRNPs and free mRNAs [61]. In the cell, the preferential membership in either group of mRNAs is probably dictated by the triggers, i.e., specific YB-1 binding sites or partners, such as RBPs or long non-coding RNAs (lncRNA) that recruit and anchor YB-1 onto certain mRNAs. In specific conditions, hundreds of mRNAs are found to be YB-1-inhibited, but the inhibitory mechanism remains obscure. It is possible that specific YB-1 binding sites (e.g., those in *YB-1*, *protamine*, etc. mRNAs [1]) ensuring an elevated YB-1 binding capacity play the role of such a trigger. The same is probably true for the translation inhibition of nodal mRNAs (*sqt*, *nodal*, *lefty*, etc.) through YB-1 binding to the DLE motif (AGCAC followed by a short hairpin) within the 3′UTRs [107]. In the case of senescence-associated mRNAs (*IL8*, *CXCL1*, etc.), YB-1 also inhibits their translation through binding to the 3′UTRs, yet it is unclear whether this binding is specific and direct [72]. 

The packaging hypothesis proposing a mechanism of translation inhibition by YB-1 is not the only one discussed in the literature. Alternatively, it may be the ability of the cold shock domain to displace eIF4E from the cap structure [75] and the ability of the C-terminal domain to displace eIF4G from mRNA [108]. Since the isolated CTD-to-RNA binding is probably non-cooperative, the C-terminal domain is unable to package mRNAs into stable mRNPs [61], thereby providing non-specific translation inhibition *in vitro*. On the other hand, *in vivo*, it is full-length YB-1, but not CTD, which inhibits the translation of the reporter *IL8* mRNA [72]. This observation can be interpreted in two ways. One is that the YB-1 cold shock domain is necessary for translation inhibition through the formation of stable untranslated mRNP complexes on specific mRNAs [61]. The other is that the C-terminal domain has predominantly nuclear (not cytoplasmic) localization [56,61] that prevents its influence on translation. The involvement of the CSD itself in translation inhibition is also uncertain. It was shown that the cold shock domain alone, as well as truncated YB-1 (the 1–180 sequence containing the A/P domain, CSD, and a small fraction of CTD), produces only a slight inhibitory effect on translation [32]. Moreover, YB-1 (1–180) forms no compact mRNP complexes but only linear filaments [32]; therefore, the cold shock domain alone is not sufficient to form correct mRNP complexes.

Of note, the CSD ability to displace eIF4E is thought to be crucial for the translation of “weak” mRNAs, including growth-related ones [75]. The hypothesis was that “weak” mRNAs are extremely sensitive to the eIF4E concentration, whose minor decrease can cause translation inhibition [75]. Indeed, the Ribo-Seq (Ribosome profiling) data confirm that mRNAs with long 5′UTRs, including growth-related ones, are extremely sensitive to eIF4E [109]. With only a small fraction of eIF4E-dependent mRNAs controlled by YB-1, the selectivity in translation inhibition by YB-1 cannot be attributed to eIF4E. The translation inhibition of tumor- and growth-related mRNAs (*CCND1, CCNE1*, etc.) was shown to depend on the YB-1 phosphorylation status [75,107]. Even though *in vitro* p-Ser102-YB-1 has the same RNA binding efficiency as unphosphorylated YB-1 [75], it is only the unphosphorylated YB-1 that can efficiently bind *in vivo* to growth-related and tumor-related mRNAs [75,107]. It can be suggested that binding to some unknown YB-1 partner acting as a trigger in the packaging of these mRNAs into untranslated mRNPs is phosphorylation-dependent. 

In total, we speculate that for the correct specific translation inhibition by YB-1, both the CSD and CTD domains are required; and perhaps the packaging mechanism is the only one realized in both germ and somatic cells.

### 5.2. YB Proteins in Translation Stimulation

As was discussed previously, YB-1 acts as the general and specific translation inhibitor for hundreds of mRNAs; hence, an increase in global translation may be expected upon YB-1 knockout. Quite the opposite, YB-1 knockout produces a slight decline in global translation in mouse and human cells [79,103]. This fact can be explained as follows. First, other RNA-binding proteins, such as the homolog YB-3, can be overexpressed in YB-1-null cells and compensate for the YB-1 absence [79]. Second, some other compensatory mechanisms can be turned on, such as an increased mRNA decay or activated signaling cascades of translation regulation. Lastly, since at a low YB-1/mRNA ratio YB-1 can activate translation *in vitro*, its knockout can lead to a decrease in the global translation. Of note, in a cell-free translation system (CFTS), YB-1 stimulates translation inhibited by excess mRNA [94]. It was proposed that mRNA-bound YB-1 displaces and accumulates translation initiation factors [94]. This mechanism probably could be critical in starvation and stress conditions accompanied by the sequestration of translation factors. Nearly the same mechanism was proposed to explain how general RBPs, including YB-1 increase the specificity of translational control [110]. For example, in CFTS, PABP and poly(A)-tail stimulate translation only in the presence of YB-1 [111]. Moreover, *in vivo*, YB-1 can influence PABP (PABPC1) localization. PABPC1 is a shuttle protein with predominantly cytoplasmic localization that binds to mRNA after its export from the nucleus [112]. Recently, it was found that PABPC1 accumulates in the nucleus upon YB-1 knockdown [113]. We speculate that YB-1 creates not only competitive conditions favorable for PABP functioning but also assists mRNP rearrangement in the nuclear pore. This idea was supported by the perinuclear localization of YB-1 [114] and its direct binding to PABP [86]. 

To date, there is no direct evidence for the stimulation of the global translation by YB-1 *in vivo*. It was found that YB-1 stimulates the translation of epithelial-mesenchymal transition- (EMT) and stress- -related mRNAs (*Snail1*, *HIF-1α*, *G3BP1*, *MYC*, etc.) predominantly in conditions of starvation or hypoxia [72,115,116,117]. The mRNAs of this class usually harbor highly-structured 5′UTRs [117]. Even though these findings were reported 10 years ago and amply confirmed in the literature [72,118,119], nearly nothing is known about the molecular mechanism. One of the viewpoints is that YB-1 unwinds the secondary structure [70] and promotes the scanning. The 48S pre-initiation complex is shown to accumulate in YB-1-depleted CFTS [120], which is evidence for YB-1 involvement in the mRNA scanning. Another idea is that the mRNAs of this class harbor an IRES (internal ribosome entry site) that is activated by YB-1 functioning as an ITAF (IRES trans-acting factor) [115]. However, there is no conclusive [121] evidence for IRES activity in these mRNAs. In contrast, the best-studied *HIF-1α* mRNA showed no IRES in the 5′UTR [122,123]. Some other proteins, such as HuR, PTB, RBM38, etc., bind to the *HIF-1α* 5′UTR and affect the translation of this mRNA [124]. Presumably, YB-1 promotes RBP binding either by the local structure unwinding or through the direct interaction with the protein in question. For some mRNAs, such as that of *G3BP1*, the data on YB-1 involvement in translational control are contradictive. Some researchers report that YB-1 enhances *G3BP1* mRNA translation through binding to the 5′UTR [125,126], while others deny such regulation [127,128]. The difference in conclusions can be explained by the presence or absence of regulatory RBPs whose binding and activity are modulated by YB-1. Another example is the stimulation of the translation of self-renewal and proliferation mRNAs (*CDK1*, *CDK2*, *EZH2*, etc.) in progenitor cells [74] where the YB-1–DDX6 complex interacts with a stem-loop structure within the 3′UTR and recruits eIF4E, which stimulates the translation of target mRNAs [74].

Thus, the YB-1 stimulating activity in translation is shown and proved both *in vitro* and *in vivo*. However, further extensive studies are required to decipher the molecular mechanism underlying YB-1-mediated translation stimulation.

## 6. YB Proteins in mRNA Stability Control

YB proteins are involved in stability and decay regulation, both globally and for specific mRNA groups. They form compact saturated mRNPs with inaccessible 5′ and 3′ ends, thereby not only inhibiting translation but also stabilizing mRNAs [101]. This mechanism is very important in germ cells, especially oocytes. The knockout of the major germ-specific mouse MSY2 protein leads to mRNA destabilization, and hence, to the arrest of oocyte growth and maturation [129]. In oocytes, MSY2 packages mRNAs into highly compact (Triton X-100 insoluble) mRNP complexes inaccessible for the degradation machinery of the cell. During oocyte maturation, MSY2 is phosphorylated by CDK1 to induce the transition of MSY2-containing mRNPs to less compact (soluble) complexes, thereby triggering mRNA translation and decay [130]. Of note, the maternal mRNA clearance happens in several waves. The exact mechanism of mRNA clearance wave-specificity is unknown, but YB proteins are supposedly involved in this process. The literature presents several recent reports about zYB-1 involvement in mRNA stability and decay regulation during the maternal-to-zygote transition (MTZ) [86,102]. Surprisingly, no global mRNA destabilization was observed in zYB-1-null oocytes [102], even though there are no other YB proteins in zebrafish. Instead, an impaired decay of unstable maternal mRNAs [102] and an elevated decay of stable mRNAs [86] were found. In zebrafish, a group of unstable mRNAs degrades mostly by the miR-430-dependent pathway [131]. It is known that YB-1 regulates miRNA processing [77]. Therefore, we can speculate that zYB-1 enhances miR-430-dependent maturation or participates in miRNA-dependent mRNA decay through its unwinding or annealing activity. On the contrary, maternal mRNAs showing relative stability during MTZ are not targeted by miR-430 and undergo m^5^C modification [86]. The zYB-1 protein binds to these mRNAs and stabilizes them, probably by PABP recruiting [86]. Interestingly, a similar process was observed in human urothelial carcinoma of the bladder, where an elevated level of m^5^C modification results in elevated YB-1-dependent stability of appropriate mRNAs [132]. The authors proposed that YB-1 recruits to this process ELAVL1 (HuR), which is another stabilizing RBP [132]. 

In somatic cells, YB proteins are involved in the regulation of decay of mRNAs containing specific stabilizing or destabilizing sequences in complexes with other RBPs. For example, immune response-related mRNAs (*CCL2*, *CCL7*, *BCL3*, etc.) have a GC-rich specific sequence within their 5′UTR that binds the glucocorticoid receptor (GR). After stimulation, GR recruits the PNRC2–UPF1–Dcp1 complex that, in turn, recruits YB-1 and HRSP12, thus triggering rapid mRNA decay [133,134]. All RBPs are required for mRNA decay [133,134], but only the decapping enzyme (Dcp1) and the endonuclease HRSP12 initiate the decay directly. The exact role of other RBPs, including YB-1, is unknown. The stability of *β-globin* mRNA is controlled by the CUGGG element within the 3′UTR [135]. AUF1 and YB-1 bind this element and enhance PABP binding to *β-globin* mRNA, which provides the stabilization of this mRNA [135]. 

In other cases, YB-1 involvement in mRNA stabilization is probably realized through preventing the binding of destabilizing AU-rich elements (AREs) within 3′UTRs to RBPs triggering mRNA decay. Interestingly, the regulatory elements responsible for YB-1 binding can localize to 5′ and 3′ UTRs and contain no specific YB-1 binding site. For example, the 5′UTR of *IL-2* mRNA contains a JNK (c-Jun N-terminal kinase) response element, which binds YB-1 and nucleolin and protects the *IL-2* mRNA from ARE-mediated decay [136]. Upon JNK activation, NF-90 transfers to the cytoplasm, binds to AREs, and stabilizes the *IL-2* mRNA [137]. YB-1 and nucleolin possibly promote the NF-90 binding. The complexes stabilizing this mRNA can comprise additional proteins. As shown, the complexes assembling on *IL-2-* and *VEGF* mRNA stabilizing elements are similar and may contain PTB and some other unidentified proteins [64]. In the case of *GM-CSF* mRNA, YB-1 along with hnRNPC and another stabilizing ARE-binding protein ELAVL1 (HuR) bind to AREs within the 3′UTR upon TNF-α (tumor necrosis factor) and fibronectin treatment [138,139].

The coding region determinant (CRD) is another mechanism that controls mRNA stability. In contrast to the above examples, the translating ribosome displaces a large protein complex from the CRD, thereby triggering mRNA decay. In the case of *c-Myc* mRNA, the protein complex consists of IGF2BP1 (IMP), YB-1, hnRNPU, DHX9, and SYNCRIP [140]. Of note, IGF2BPs bind the GGm^6^AC motif at the end of the coding region (probably at the CRD) and additionally recruit ELAVL1 (HuR) and PABP that stabilize mRNAs [141]. As follows from the number of mRNAs bound to IGF2BPs, the described mechanism is rather widespread. However, the exact role of YB-1 in this process is unknown. Recently, it was found that even though YB-1 and IGF2BP1 do not interact directly, they bind the same mRNA molecules immediately after mRNA nuclear export [142]. 

Similar to YB-1, YB-3 can participate in mRNA stability control. YB-3 can bind 3′UTRs of *p21* [143], *SLC7A5*, and *SLC3A2* (amino acid transporters) mRNAs and stabilize them [78]. However, nothing is known about the mechanism.

Thus, even though YB-1 and YB-3 are essential for the stability and decay control of certain mRNAs, nearly nothing is known about their exact role in this process. Supposedly, due to multiple protein partners and the RNA unwinding ability, YB proteins facilitate the formation of correct mRNP complexes regulating mRNA stability and decay.

## 7. Modulating Activity of YB Proteins in Translation and Stability

YB-1 activity in translation and stability can be modulated by YB-1 modification (Table 1), non-coding RNAs (Table 2), or subcellular localization. For example, YB-1 phosphorylation at Ser102 promotes the translation of growth-related mRNAs [75,107], while the overexpression of YB-1 is unable to be phosphorylated at Ser102 and promotes the translation of EMT-related genes [144]. YB-1 acetylation at Lys81 prevents *HIF-1α* and *G3BP1* translation activation [125]. An unknown modification of YB-1 (possibly phosphorylation) upon serum or IGF-I stimulation decreases the ability of YB-1 to bind *OXPHOS* mRNAs (*NDUFA9*, *NDUFB8*, *SDHB*, etc.), thereby promoting their recruitment to polysomes [145].

The YB-1-binding lncRNAs (Table 2) exclude YB-1 from the translation/stability regulation and affect its localization or stability. Another intriguing possibility is that YB-1 assists lncRNA in translational control. For example, lnc-31 not only stabilizes YB-1 but also, in concert with it, binds to *ROCK1* mRNA to activate its translation [146]. It is unclear whether the effects of YB-1 and lnc-31 are cooperative or independent. The amount of YB-1 can be modulated by miRNAs through the regulation of *YB-1* mRNA translation or decay [147,148,149,150]. Moreover, miRNAs can compete with YB-1 for binding to specific sequences within mRNAs that interfere with translational or stability control. For example, in chondrocytes, a mutant of miR-140-5p competes with YB-1 for YB-1-specific binding sites within the 3′UTRs, which decreases the mRNA stability [151]. 

The YB-1 involvement in translation or stability depends on cellular conditions. For example, with simultaneous unfolded protein response (UPR) and hypoxia, YB-1 fails to stimulate *HIF-1α* mRNA translation [152]. The authors explain this fact by a decreased YB-1 ability to bind the 5′UTR of *HIF-1α* mRNA [152]. However, there is another explanation. Under UPR, YB-1 localizes to stress granules (SG) formed within the cell [127], and hence, the cytoplasmic free YB-1 is insufficient for stimulation of *HIF-1α* mRNA translation.

**Table 1:** Modifications of YB proteins and probable effects on their functions. The complete list of detected modifications can be found at www.phosphosite.org. The names of respective YB proteins except YB-1 (default) are shown in bold in parentheses. Notation: ↑—up-regulation; ↓—down-regulation; n/e—no effect; ?—indirect evidence; ??—unknown function.

Modification	Location	Enzyme	Probable Effect	Reference
*Acetylation*				
Lys81	CSD	HDAC1 (?) HDAC3 (?)	YB-1 binding and translational activation of *G3BP1, NFE2L2, HIF-1α* mRNAs (↓)	[125]
Lys 303, Lys304	CTD	??	YB-1 secretion (↑)	[153,154]
*O-GlcNAcylation*				
Ser32	A/P	OGT		[155]
Thr126	CSD		Ser102 phosphorylation (↑)	
Ser209, Ser313	CTD			
*Phosphorylation*				
Thr58, Thr67, Thr78 **(MSY2)**	CSD	CDK1	RNA or mRNP protein binding (↓)	[130]
Tyr99	CSD	Akt/PI3K pathway (?)	??	[156,157]
Ser99 **(chk-YB-1)** **(Ser102 analog)**	CSD	Akt	YB-1 (Ser99E, Ser99A) translation inhibition (↓) Nuclear translocation (n/e)	[158]
Ser102	CSD	Akt RSK (CN, calcineurin dephosphorylates)	Changes in YB-1-mRNA binding targets	[75,107]
			YB-1 binding to cap-adjusted mRNA regions (↓) Nuclear translocation (n/e)	[75]
			Nuclear translocation (↑)	[159,160,161]
			Promoter binding, incl. *CCL5, EGFR, HER2* (↑?)	[159,162,163]
Ser134 **(YB-3)** **(S102 analog)**	CSD	Akt, RSK	??	[164]
Ser165	CTD	CKII (?)	Nuclear translocation (↑) Activation of NF-κB(↑?)	[165,166]
Ser176	CTD	CKI	Nuclear translocation (↑) Activation of NF-κB(↑?)	[167]
Thr188	CTD (NLS-2)	??	??	[168]
Thr281	CTD (NLS-3)	??	Nuclear translocation (↑?)	[168]
*Poly(ADP-ribosyl)ation*				
??	CTD (219-324)	PARP1, PARP2	DNA binding (↓) APE1 activity (↑)	[169,170]
*SUMOylation*				
Putative DSKA(287-290), TKED 60-63, EKRE (151-154) **(zYB-1)**	??	??	Nuclear translocation (↑?)	[171]
*Ubiquitination*				
Lys27	A/P	HACE1	YB-1 secretion (↑)	[172]
Lys48	A/P	?? (OTUB1, deubiquitinase)	YB-1 protein stability (↓)	[173]

**Table 2:** YB-1-interacting non-coding RNAs and their probable effects on YB-1 functions. Notation: ↑—up-regulation; ↓—down-regulation; ?—indirect evidence; ??—unknown function.

ncRNA	Probable effect	Reference
*Long non-coding RNAs*		
AWPPH	Recruits YB-1 to *PIK3CA* promoter, *SNAIL* mRNA (?)	[174]
BDLNR	Recruits YB-1 to *PIK3CA* promoter	[175]
BX111887	Recruits YB-1 to *ZEB1*promoter (?)	[176]
CAR10, HNSCR	YB-1 stability (↑)	[177,178]
GAS5, MIR22HG	YB-1 stability (↑) Nuclear translocation (↑)	[179,180,181]
H19	Nuclear translocation (↑?) Recruits YB-1 to *COL1A1* promoter (?)	[182]
HITT	Sequestrates YB-1 from *HIF-1α* mRNA	[183]
HOXC-AS3	Recruits YB-1 to promoters of YB-1 and HOXC-AS3 target genes (?)	[184]
HULC	YB-1 phosphorylation at S102 (↑)	[185]
LINC00312, LINC02527	??	[186,187]
Lnc-31	YB-1 stability (↑) *ROCK* mRNA translation (↑), Recruits YB-1 to *ROCK*mRNA (?)	[146]
POU6F2-AS2	Recruits YB-1 to DNA damage sites and target promoters (?)	[188]
SCAT7	Recruits YB-1/hnRNPK to SCAT7 target promoters, including *FGFR2/3*promoters	[189]
TP53TG1	Nuclear translocation (↓)	[190]
*Circular RNAs*		
CircAnks1a	Nuclear translocation (↑), Enhances transportin 1 binding (?) Recruits YB-1 to *VEGFb* promoter	[191]
CircFAT1(e2)	Sequestrates YB-1 in nucleus (?)	[192]
CircNfix	YB-1 stability (↓) Nuclear translocation (↓)	[193]
*Satellite non-coding RNAs*		
MajSAT	Nuclear translocation (↓)	[194]
*MicroRNAs*		
miR-29b	YB-1 suppresses miRNA maturation	[77]
miR-30c, miR-320 family, miR-768-5p, miR-886, miR-923, miR-1308, miR-1973, miR-1979, miR-4284, let-7 family	??	[195]
miR-144, miR-223	YB-1 sorts microRNAs into exosomes	[98]
*Other small non-coding RNAs*		
5′-tiRNA^Ala^, 5′-tiRNA^Cys^	SG formation	[127]
*C/D Box snoRNA* (SNORD29, SNORD34, SNORD68, SNORD33), *H/ACA Box snoRNA* (ACA44)	??	[195]
mt-tRNA	??	[196]
short RNAs (shyRNAs), small RNAs (smyRNAs)	??	[197]

Changes in the subcellular distribution of YB-1 can be crucial for YB-1 availability in the cytoplasm. YB-1 can be secreted from the cell [198], localized to different RNA granules (processing bodies and stress granules) [97], or shuttle between the nucleus and the cytoplasm [1]. Nearly nothing is known about how YB-1 secretion affects its intracellular concentration. In the majority of YB-1 secretion studies, the intracellular YB-1 level was not measured [198,199,200]. Only one study reports that under oxidative stress caused by sodium arsenite or H_2_O_2_ treatment, YB-1 can secrete from the cell, and its intracellular concentration is slightly decreased [113]. Changes in the intracellular YB-1 content under oxidative stress and other stimuli are subject to further detailed investigation.

### 7.1. YB-1 in Stress Granule Assembly

YB-1 is a marker of stress granules (SG) and processing bodies (PB) [97]. Stress granules have stable core components inside a dynamic outer shell [201]. It is believed that both specific RNA-protein and protein–protein interactions, as well as non-specific interactions between intrinsically disordered regions of proteins, drive the liquid–liquid phase separation that causes a spontaneous SG core assembly [201]. Since YB-1 can oligomerize and interact with multiple protein partners and more than half of its molecule is intrinsically disordered (see above), this protein may be involved in SG assembly and formation of the SG core. According to [126], YB-1 overexpression causes no SG assembly; hence, YB-1 hardly acts as a nucleator or resides within the SG core. Moreover, in many conditions, YB-1 is not essential for SG assembly. Its knockout or knockdown does not prevent p-eIF2α-dependent SG formation under UPR and oxidative stress [126,127,202]. On the other hand, the non-canonical (p-eIF2α-independent) SG assembly induced by tiRNA (tRNA-derived stress-induced RNA) is reported to be strongly influenced by YB-1 [127]. This discrepancy could be attributed to the different mechanisms of SG formation. The data concerning the YB-1 effect on SG assembly are also quite contradictory. Some studies have demonstrated that YB-1 knockout or knockdown results in a lower number of SG-positive cells [126,127], whereas, according to other reports, YB-1 knockdown causes no change in the number of SG-positive cells but provokes an increase in SG number and a decrease in SG size [202]. These observations can be cell type- and experiment-specific. For example, the observed effect depends on arsenite concentration [127] and can rely on the expression of other SG promoting proteins, especially in knockdown experiments. The decreasing size of SGs can be attributed to the tubulin-binding ability of YB-1 [203]. As was shown, the intact microtubules are crucial in a fast aggregation of small SGs into larger ones [204]. In addition to the data on the SG assembly-promoting activity of YB-1, it was shown that YB-1 overexpression prevents SG assembly [126,202] and that YB-1 can disassemble large FUS- and TDP-43 mRNP aggregates with the formation of its own mRNP particles [205]. We speculate that elevated YB-1 concentration leads to the formation of mRNP complexes inaccessible to other SG proteins. For example, YB-1 protects *Hsp70* mRNA from recruitment to SGs upon arsenite treatment, whereas a decreased YB-1 level causes *Hsp70* mRNA localization in SG and translation inhibition [202]. Thus, the exact role of YB-1 in SGs is unknown. We propose that YB-1 may serve as an mRNA-sorting factor depending on a particular mRNP complex. Interestingly, YB-3 has been recently detected in SG [206], although nothing is known about its function in this context.

### 7.2. Nuclear-Cytoplasmic Transport of YB-1

YB-1 is a nuclear–cytoplasmic shuttling protein whose subcellular localization is dictated by the nuclear localization signal (NLS, 186–205 a.a.) and the cytoplasmic retention signal (CRS, 267–293 a.a.) [56,207]. The YB-1 nuclear localization signal is considered as a PY-NLS recognized by transportin 1 [208,209]. Some other motifs, such as NLS-1 (149–156 a.a.), NLS-2 (185–194 a.a.), and NLS-3 (276–292 a.a.), can also influence YB-1 localization [168]. It is believed that CRS prevails over NLS and causes the basically cytoplasmic YB-1 localization. The 20S proteasome cleaves off a C-terminal part of YB-1 (220–324 a.a.), thereby triggering the nuclear accumulation of the truncated protein [210]. Along with CRS, the intact CSD is also responsible for YB-1 retention in the cytoplasm. Although CTD contains CRS, it localizes to the nucleus [56,61,168]. Moreover, mutant YB-1 within RNP consensuses of CSD (Phe85Ala, Tyr72Ala+Phe74Ala) is also localized to the nucleus [127,207]. It is unknown how mutations in RNP consensuses influence the CSD structure and stability; therefore, it is difficult to conclude whether the CSD itself or its RNA-binding ability is responsible for YB-1 retention in the cytoplasm. It is a recent finding that long-lasting transcription inhibition causes mRNA degradation and promotes YB-1 accumulation in the nucleus [211,212,213,214]. Of note, upon transcription inhibition, not only the cytoplasmic mRNA content but also YB-1 modifications play a key role in the YB-1 nuclear import [211]. The best-studied YB-1 modification is its phosphorylation at Ser102 by Akt or RSK [75,161,162], which promotes YB-1 nuclear accumulation [159,160,161] by an unknown mechanism. YB-1 undergoes some other modifications (Table 1) that can also modulate its RNA-binding activity and/or nuclear–cytoplasmic transport. 

Nearly nothing is known about YB-1 nuclear export. Despite the predicted classical nuclear export signal (NES), YB-1 nuclear export is exportin 1 (CRM1)-independent [168,207]. Possibly, YB-1 binds mRNAs immediately after or co-transcriptionally, and its translocation to the cytoplasm occurs in concert with mRNAs and mediates by RNA export proteins. This hypothesis is indirectly supported by two facts: fast YB-1 export after the cessation of the transcription blockage [211] and YB-1 association with chromatin [89,90] suggesting co-transcriptional YB-1 binding to nascent RNA. 

## 8. Conclusions

This review presents new data on the YB-1 structure, RNA binding, and the mechanisms of involvement of YB proteins in the regulation of mRNA translation and stability. Despite the progress in these fields, there are numerous gaps in our knowledge. The structure of full-length YB-1 is still a mystery. We do not know the effect of RNP consensus mutations on CSD stability and structure. There are questions as to YB-1-RNA interactions, especially the role of the C-terminal domain and mRNP assembly in the presence of other RBPs. The exact mechanisms of YB-1-dependent selective translational and stability controls are studied only partially, especially *in vivo*. Nearly nothing is known about the role of YB-1 in SG assembly and functioning.The mechanisms of YB-1 nuclear export, as well as those of RNA binding and phosphorylation involvement in YB-1 nuclear import, are obscure.

Our knowledge of other YB proteins is very far from comprehensive. In comparison with YB-1, we know little about the role of YB-3 in SG and mRNP assembly, translational, and stability controls, as well as its nuclear-cytoplasmic translocation. Since YB-1 and the long isoform of YB-3 are close in structure and sequence, the latter might similarly shuttle between the nucleus and the cytoplasm and replace YB-1 in the regulation of mRNA translation and stability. As shown, the synthesis of YB-3 is up-regulated in YB-1-null cells [79]; therefore, the functional interchangeability of these proteins should be taken into account when interpreting experimental data, especially those on YB-1 knockout and knockdown.
